# Effectiveness of Hospital–Community Partnerships in Preventive Health Care Interventions: An Exploration of Racial and Ethnic Disparities in Impact

**DOI:** 10.1089/heq.2023.0259

**Published:** 2025-01-08

**Authors:** Ohbet Cheon, No Young You

**Affiliations:** ^1^ David D. Reh School of Business, Clarkson University, Schenectady, New York, USA.; ^2^ New York State Department of Health, Bureau of Early Intervention, Albany, New York, USA.

**Keywords:** hospital-community partnership, vaccination, mammography, preventive care, racial-ethnic disparities, population health

## Abstract

**Introduction:**

Hospital–community partnerships have been increasingly emphasized to improve population health in recent decades. This study investigates the effectiveness of hospital–community partnerships in preventive health care interventions, addressing potential racial and ethnic disparities in impact.

**Methods:**

We measured overall hospital–community partnerships with nine community organizations at the county level using the American Hospital Association annual survey. Preventive health care interventions were also measured by preventable hospitalization rates, mammography screening rates, and flu vaccination rates across racial and ethnic groups using County Health Ranking National data. We estimated pooled ordinary least squared models with year-fixed effect and robust cluster standard errors at the state level. We also used generalized least squares models to examine the impact across racial and ethnic groups, including controls for county characteristics.

**Results:**

Among 3785 counties across 50 states in the United States in the pooled data, the findings indicated that hospital–community partnerships were effective in increasing mammography screening and flu vaccination rates in general. However, upon closer examination of the impact across racial and ethnic groups, hospital–community partnerships were not significantly associated with any of the interventions in the Black and Hispanic population, while they were effective in the White population.

**Discussion:**

Hospital–community partnerships can be effective in increasing uptake rates for mammography screening and flu vaccination rates, but their impact is unevenly distributed among racial and ethnic minorities.

**Health Equity Implications:**

The findings emphasize the need to design targeted hospital–community partnerships for racial and ethnic minorities to mitigate health disparities in preventive health care interventions.

## Introduction

Hospital–community partnerships have garnered significant attention from policymakers and health care leaders to improve population health in recent decades. With the implementation of the Affordable Care Act (ACA) in 2010, hospitals have been incentivized to establish strategic partnerships with community organizations, initially focusing on enhancing the quality and cost of care.^1–4^ As the health care paradigm has shifted from curative care to preventive care, hospital–community partnerships are more encouraged to enhance care coordination, enable health services for preventable diseases, and address social determinants of health for patients.^5,6^ The Robert Wood Johnson Foundation Commission to Build a Healthier America has also emphasized the roles of community stakeholders—such as local health agencies, social services, nonprofits, and faith-based organizations—and recommended active collaboration with these entities to foster health-promoting behaviors.^7,8^ Among stakeholders, the nationwide American Hospital Association (AHA) Population Health Survey revealed that local health departments are the most important key partner to improve population health.^9^ Other studies also supported that hospital-public health collaboration contributes to healthier communities.^10–12^

While hospital–community partnerships hold promise to improve population health, it remains understudied whether such partnerships are effective in preventive health care interventions. Many studies have focused on the impact of these partnerships on mortality,^13^ readmissions,^4^ ED and urgent care visits,^14^ and cost of care^15,16^ at the hospital-levels. With the focus on preventive care under the ACA and Community Health Needs Assessment (CHNA) implementation,^17^ hospital–community partnerships are viewed as key factors in improving preventive health care interventions, including vaccinations, cancer screenings, and health education at the community level. According to the AHA population health survey, around 70% of hospitals reported that they are part of a communitywide coalition for preventive care.^9^ More specifically, according to the AHA annual survey in 2016, about 74% of hospitals provided mammography screening, and 44% of hospitals offered immunization programs at their facilities or their subsidiary.^18^ Beyond traditional settings, hospital–community partnerships can establish navigator programs that raise awareness of cancer preventive care throughout health care delivery.^19,20^ Existing studies also support that targeted inpatient mammography screening can effectively enhance access to preventive care by addressing challenges related to scheduling, keeping appointments, transportation, and taking time off from work.^21^ Additionally, hospitals can use their patient portals to promote patient engagement in preventive interventions, such as flu vaccinations, especially for patients at risk of other diseases like asthma.^22^

However, a few case studies have explored specific hospital–community partnership programs, examining their effectiveness on mammography uptake,^23,24^ flu vaccinations,^25^ and COVID-19 vaccinations^26^ in certain regions, which underscores the need to generalize their impacts using nationwide data. Rather focusing on a specific partnership or program, this study aims to examine the outsized impact of overall hospital–community partnerships on preventive health care interventions at the county level across 50 states in the United States. With this similar macros perspective and methods, previous studies also have explored the impact of such hospital–community partnerships on COVID-19 case-fatality rates^27^ and ambulatory care sensitive condition prevalence^28^ at the hospital service areas.

In addition, this study investigates whether the effectiveness of hospital–community partnerships varies among different racial and ethnic groups. Previous research suggests that these partnerships may reduce health disparities by facilitating community engagement and identifying the unmet needs of minorities.^13,29,30^ However, other studies raise concerns that hospital-community partnerships may disproportionately benefit historically advantaged groups who have fewer barriers, such as English-language users.^22^ Given the significant disparities in preventive health care interventions among racial and ethnic groups, it is crucial to explore whether the disparities can be reduced by hospital–community partnerships. Studies found that African American women are less likely to uptake mammography screening compared with White women, resulting in significantly higher breast cancer mortality rates at every age.^23^ Furthermore, Black and Hispanic children are less likely to receive flu vaccines compared with their White counterparts.^31,32^ This disparity can be attributed, in part, to higher vaccine hesitancy, driven by ongoing access and cost barriers, as well as medical mistrust within minority communities.^25^ Studies have also found that African Americans and Hispanics experience significantly higher rates of ambulatory care sensitive hospitalization than non-Hispanic Whites, primarily due to the lack of access to primary care and preventive interventions.^33,34^ These studies highlight the racial and ethnic disparities in preventive health care interventions and underscore the need to explore the impact of hospital–community partnerships on these disparities.

## Methods

### Data

This study mainly uses three nationwide datasets: the 2016 and 2017 AHA annual survey, the 2020 and 2021 County Health Ranking National (CHRN) dataset, and the 2018 Geography of Social Capital Data. The AHA data provided hospital-level community partnerships across nine community organizations in fiscal years (FY) 2016 and 2017. The CHRN dataset published in 2020 and 2021 collected various county-level sub-datasets measured between 2017 and 2019, such as the Mapping Medicare Disparities (MMD) Tool, American Community Survey (ACS) (5-year estimates), Area Health Resource File, Small Area Health Insurance Estimates, and Census Population Estimates. The Geography of Social Capital Data provided the county-level social capital index, which allows us to compare the relative impact of social capital across counties.

Considering hospital–community partnerships in FY2016 and 2017 AHA surveys, these county-level measures in 2020 and 2021 CHRN reports are well-matched for assessing the association of such partnerships with preventive health care interventions as outcome measures. Moreover, our analysis in 2016–2019 can be important to demonstrate the impact of hospitals’ partnership efforts before the onset of the global pandemic in their communities. Lastly, since the CHNA requirement for tax exemption was implemented in December 2015,^35^ the year of 2016–2019 analysis underscored how hospitals initially promote community partnerships under the ACA regulation and their impacts on health disparities.

**FIG. 1.**
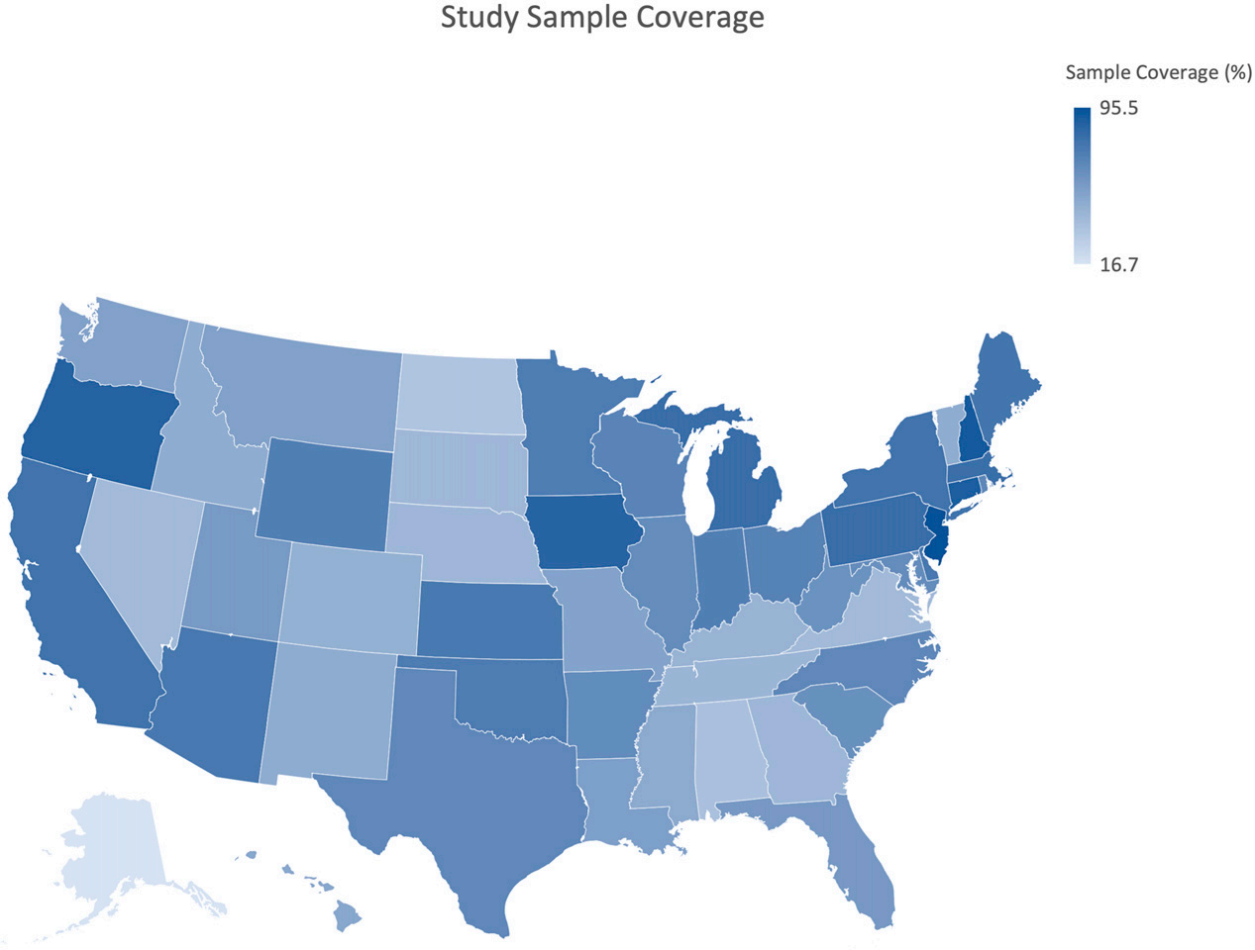
The study sample coverage across states.

To create the study sample, this study initially considered all U.S. counties in CHRN data (total *N* = 6286; 3143 in each year) as the study population, and then refined this selection to include only counties with hospitals participating in the AHA annual survey in FY2016 and 2017 (total *N* = 3785; 1892 in 2016 and 1893 in 2017). The hospital-level AHA data were merged with county-level CHRN data using county codes to match hospitals with their respective counties, and the average hospital–community partnership was calculated for each county. Since hospitals typically choose local community organizations as partners,^36^ this approach allowed us to assess the impact of hospital–community partnerships on preventive health care interventions within their primary county. Additionally, this study accounted for various county-level variables that might influence preventive health care interventions. Following the merger of the three datasets and the elimination of missing observations, the pooled dataset encompassed 3785 counties across 50 states and Washington D.C. Figure 1 visualized the percentage of counties covered by the study sample across states, with darker areas indicating greater coverage. In general, the study sample covers more than 59% of U.S. counties across states.

### Measures

Using the MMD Tool in CHRN data, this study focuses on three preventive health care interventions as dependent variables: preventable hospitalization rate, mammography screening rate, and flu vaccination rate at the county level. Based on Medicare enrollees, the MMD Tool data allows comparison of preventive intervention rates using standardized measures across counties. The preventable hospitalization rate is a rate of hospital stays for ambulatory care sensitive conditions per 100,000 Medicare enrollees, adjusted for age and sex. A higher value indicates a worse outcome. Ambulatory care sensitive conditions are defined as conditions for which outpatient care or early interventions can possibly prevent hospitalizations, so this study incorporates this measure as an outcome reflecting the lack of preventive health care interventions. This study also measures two uptake rates for preventive services. The mammography screening rate is measured as a percentage of female Medicare enrollees ages 65–74 who received an annual mammography screening. And the flu vaccination rate is measured as a percentage of fee-for-service Medicare enrollees who had an annual flu vaccination. All three outcomes are measured by race and ethnic groups separately, such as Black, Hispanic, and non-Hispanic White. The MMD Tool data included American Indian/Alaska Native (AIAN) and Asian groups, but these groups were excluded from our analysis due to a high rate of missing observations (about 80% of our sample) at the county level.

The key independent variable is the hospital–community partnership. The 2016 and 2017 AHA annual survey asked hospital managers about *the extent of your hospital’s current partnerships with the following nine types of organizations for community health improvement initiatives*. The response rate was about 59%, indicating that more than 3000 hospitals across counties responded to this questionnaire. The nine community organizations include (1) health care providers outside their hospitals, (2) local public health organizations, (3) local social service agencies, (4) local/state governments, (5) nonprofit organizations, (6) faith-based organizations, (7) insurance companies, (8) schools, and (9) local businesses. The hospital–community partnerships were categorized into three options for each partner: non-involved, collaboration (exchange information and share resources), and formal alliance (i.e., binding agreement). We measure each partnership as an ordinal variable, and then conduct polychoric principal component analysis to generate a single common component among the nine partnerships, following the approach used in previous studies.^4,37^ As this study focuses on county-level impact, we operationalize the overall hospital–community partnership in each county by calculating the average of common component partnerships across hospitals within a county. Additionally, the analysis model includes the number of hospitals in a county as a control variable.

Based on the social determinants of health model, various county characteristics are considered as control variables. Specifically, the Healthy People 2030 initiatives identify five domains of social determinants of the health model that affect community-level health initiatives, health outcomes, and health equity: economic stability, education access and quality, health care access and quality, neighborhood and built environment, and social/community context.^38^ This study controls for these domains at the county level. For economic stability, we include unemployment rate (the percentage of the population aged 16 and older unemployed but seeking work), median household income (the income where half of the households in a county earn more and half of households earn less, log transformed), and income inequality (the ratio of household income at the 80th percentile to income at the 20th percentile, log transformed). For education access and quality, we control for college degree holders (the percentage of adults ages 25–44 with some post-secondary education). In terms of health care access and quality, we control for clinical accessibility regarding preventive care, including primary care provider (PCP) ratio (the ratio of population to primary care providers, log transformed), other primary care providers (PCP) ratio (the ratio of population to primary care providers other than physicians, log transformed), and uninsured population (the percentage of population under age 65 without health insurance). As neighborhood/built environment and demographic factors, we include rurality (the percentage of population living in a rural area), population size, female (%), Hispanic (%), and Black (%) population at the county level. In terms of social/community context, we include the county-level social capital index in the model, which has been examined in conjunction with hospital–community partnerships.^39^ Previous studies indicate that high levels of social capital promote active partnerships between hospitals and community organizations, strengthening social and community support for health initiatives.^11,37^ Table 1 shows descriptive statistics, detailed measurements, and data sources of all variables.

**Table 1.** Descriptive Statistics for Variables

Variables	N	Mean	Standard deviation	Min	Max	Source
*Dependent Variables (DVs)*						
Preventable hospitalization rate	3785	4622.8	1625.2	455	16,802	Mapping Medicare Disparities Tool 2017 and 2018^a^
% With annual mammogram	3785	41.7	7.4	4	64	
% Flu vaccinated	3785	44.1	9.4	4	67	
*DVs by racial and ethnic groups*						
Preventable hospital rate (Black)	2037	6522.4	3638.2	361	55,556	
Preventable hospital rate (Hispanic)	1757	4418.4	3007.4	249	35,714	
Preventable hospital rate (White)	2618	4551.7	1556.5	870	17,008	
% Mammogram (Black)	2102	39.6	8.3	11	81	
% Mammogram (Hispanic)	2246	34.9	9.4	10	82	
% Mammogram (White)	2832	41.8	7.2	12	70	
% Flu vaccinated (Black)	2573	34.4	8.5	4	69	
% Flu vaccinated (Hispanic)	2761	37.6	9.8	4	75	
% Flu vaccinated (White)	3225	46.3	8.8	9	68	
*Independent Variable*						
Hospital–community partnership	3785	−1.9	1.3	−4.6	2.2	AHA Annual Survey 2016 and 2017
*Controls*						
The number of hospitals in county	3785	2.2	5.1	0	101	AHA Annual Survey 2016 and 2017
Rurality, %	3785	47.1	28.9	0	100	Census Population Estimates 2010^a^
Unemployed, %	3785	3.9	1.3	1.6	17.0	Bureau of Labor Statistics 2018 and 2019^a^
Median household income (log)	3785	10.9	0.2	10.2	11.9	Small Area Income and Poverty Estimates 2018 and 2019^a^
Income inequality	3785	4.5	0.7	3.0	12.0	ACS, 5-year estimates (2014–18) and (2015–19)^a^
PCP rate (log)	3785	7.5	0.5	5.2	10.8	Area Health Resource File 2017 and 2018^a^
Other PCP rate (log)	3785	7.1	0.5	4.1	9.1	Area Health Resource File 2017 and 2018^a^
Uninsured, %	3785	11.0	5.2	2.3	33.8	Small Area Health Insurance Estimates 2017 and 2018^a^
College, %	3785	60.1	11.0	19.2	91.1	ACS, 5-year estimates (2014–18) and (2015–19)^a^
Female, %	3785	50.1	1.9	29.4	56.5	Census Population Estimates 2018 and 2019^a^
Hispanic, %	3785	10.7	14.0	0.7	96.4	Census Population Estimates 2018 and 2019^a^
Black, %	3785	8.4	12.8	0.1	82.5	Census Population Estimates 2018 and 2019^a^
Population size at county	3785	73,584.7	220,473.7	233	6,886,895	Census Population Estimates 2018 and 2019^a^
Social capital index at county	3785	0.0	1.0	−3.7	2.7	The Geography of Social Capital Dataset 2018
Year	3785	2016.5	0.5	2016	2017	

^a^ These datasets are included in the CHRN data in 2020 and 2021.

### Statistical analysis

This study tested two hypotheses: (1) whether hospital–community partnership is effective in preventive health care interventions, and (2) whether there are disparities across racial and ethnic groups in its impact. For the first hypothesis, the pooled ordinary least squares (OLS) model was estimated with year-fixed effects and robust cluster standard errors at the state level, including controls for county characteristics. The models satisfied diagnosis tests for OLS assumptions. Subsequently, the study tested the second hypothesis by conducting generalized least squares (GLS) models with gamma distribution separately for Black, Hispanic, and non-Hispanic White groups. The models included year-fixed effects and robust cluster standard errors at the state level as well. The GLS models were estimated with the consideration of heteroskedasticity and skewness due to the non-normal distribution of preventive health care interventions across race and ethnic groups.

## Results

The study sample included 3785 counties across 50 states and D.C. When examining preventive health care interventions by racial and ethnic groups separately, sample sizes were slightly reduced, but most counties reported outcomes by racial and ethnic groups. The descriptive statistics indicated racial and ethnic disparities in preventive health care interventions. Table 1 shows that the Black population had the highest rate of preventable hospitalizations compared with the overall average and other racial and ethnic groups. In mammography screening, the Hispanic population had the lowest average screening rate (34.9%) compared with the Black (39.6%) and White (41.8%) population. In flu vaccination, the Black population had the lowest rate (34.4%) compared with the Hispanic (37.6%) and White (46.3%) population. Overall, the White population shows better outcomes across all three preventive interventions compared to the Black and Hispanic population. We also conducted multivariate tests of means for each preventive intervention across groups and found that there were statistically significant differences in their means across racial and ethnic groups (*p* < 0.0001). The descriptive results bring attention to further exploration into whether such disparities persist in the impact of hospital–community partnerships.

The first hypothesis was tested by estimating pooled OLS models with year-fixed effect and robust cluster standard error at the state level (see Table 2). The results showed that hospital–community partnerships were positively associated with mammography screening rate (*p* < 0.01) and flu vaccination rate (*p* < 0.01) but were not significantly associated with preventable hospitalization rate. The findings indicated that hospital–community partnerships were effective in increasing uptake rates for preventive services in general. The models included various county characteristics as controls. Among them, the preventable hospitalization rate was negatively associated with median household income but positively associated with income inequality. Among demographic factors, the percentage of the Black population was positively associated with the preventable hospitalization rate. For mammography screening, it was negatively linked with the unemployment rate, income inequality, and uninsured rate, but positively associated with the population size and Black population rate. Regarding flu vaccination, it was negatively associated with rurality, unemployment, and the uninsured rate, but positively linked to median household income and other primary care provider rates. The percentage of the Hispanic population was negatively associated with both mammography screening and flu vaccination, while the female population was only positively associated with flu vaccination.

**Table 2.** The Impact of Hospital–Community Partnership on Preventable Health Care Interventions

	(1)	(2)	(3)
	Preventable hospital rate (reversed outcome)	% Mammogram	% Vaccinated
	b (se)	b (se)	b (se)
Hospital–community Partnership	−16.962 (29.073)	0.351** (0.105)	0.399** (0.144)
The number of hospitals in county	15.535 (10.164)	0.106+ (0.058)	0.001 (0.090)
Rurality, %	−0.632 (2.893)	−0.011 (0.012)	−0.105*** (0.017)
Unemployed, %	75.089 (78.395)	−0.854* (0.339)	−1.419*** (0.324)
Median household income (log)	−976.026** (326.792)	−0.137 (1.901)	9.506*** (1.984)
Income inequality	230.491* (92.979)	−0.897* (0.440)	0.281 (0.447)
PCP rate (log)	443.020** (127.882)	−0.615 (0.350)	−0.188 (0.504)
Other PCP rate (log)	−138.640 (124.459)	−0.933 (0.589)	1.246* (0.571)
Uninsured, %	4.509 (27.190)	−0.387** (0.111)	−0.214* (0.101)
College, %	−21.134** (6.506)	0.059 (0.039)	−0.044 (0.035)
Female, %	55.198 (31.482)	0.222 (0.127)	0.697*** (0.147)
Hispanic, %	−6.689 (8.633)	−0.082** (0.027)	−0.170*** (0.028)
Black, %	17.043* (7.251)	0.078** (0.028)	−0.044 (0.034)
Social capital index	−0.000 (0.000)	−0.000** (0.000)	−0.000 (0.000)
Population size	47.778 (180.224)	1.488** (0.538)	−1.279 (0.640)
Year = 2017	−166.545*** (39.733)	0.387* (0.153)	1.042*** (0.158)
Constant	10,038.684* (3911.725)	52.631* (21.063)	−85.039*** (23.734)
*R*-squared *N*	0.199 3785	0.396 3785	0.369 3785

^*^ *p* < 0.05.

^**^ *p* < 0.01.

^***^ *p* < 0.001.

Pooled analysis using the OLS model with year-fixed effects and robust cluster standard errors at the state level.

When investigating the second hypothesis regarding whether the effect of hospital–community partnerships is disproportionately distributed across racial and ethnic groups, disparities were found in the impact (see Table 3). When all controls were included in the model, in the White population, hospital–community partnerships significantly increased mammography screening (*p* < 0.001) and flu vaccination rates (*p* < 0.05). However, these impacts were not observed in racial and ethnic minorities. No significant associations between hospital–community partnerships and preventive interventions were observed in the Hispanic and Black populations, unlike the results seen in the White population.

**Table 3.** The Impact of Hospital–Community Partnership Across Different Racial and Ethnic Groups (Black, Hispanic, and Non-Hispanic White)

	(1)	(2)	(3)
	Preventable hospital rate (Black)	Preventable hospital rate (Hispanic)	Preventable hospital rate (White)
Hospital–community partnership	0.012 (0.012)	0.004 (0.015)	−0.003 (0.008)
*N*	2037	1757	2618
	(4)	(5)	(6)
	% Mammogram (Black)	% Mammogram (Hispanic)	% Mammogram (White)
Hospital–community partnership	0.003 (0.004)	0.010 (0.006)	0.011*** (0.003)
*N*	2102	2246	2832
	(7)	(8)	(9)
	% Vaccinated (Black)	% Vaccinated (Hispanic)	% Vaccinated (White)
Hospital–community partnership	0.001 (0.005)	0.006 (0.006)	0.006* (0.003)
*N*	2573	2761	3225

All controls were included in the analysis, but not reported in this table.

^*^ *p* < 0.05.

^***^ *p* < 0.001.

Pooled analysis using the GLS model with gamma distribution, year-fixed effects and robust cluster standard errors at the state level.

## Discussion

This study examines the effectiveness of hospital–community partnerships on preventive health care interventions and explores health disparities in impact. Using nationwide county-level datasets, the findings showed that overall hospital–community partnerships were positively associated with mammography screening and flu vaccination rates in general but not significantly related with preventable hospitalization rates. Our findings have broadened the scope of previous studies,^23,25^ generalizing the effectiveness of overall hospital–community partnerships in improving the uptake of preventive interventions across more than half of counties in all 50 states. Additionally, the results highlight the outsized effect of hospital–community partnerships at the county level, emphasizing their positive impacts on preventive interventions. Given that numerous studies have explored the impact of these partnerships on hospital- or individual-level health outcomes,^13,40^ our findings contribute to the literature by guiding the understanding of their effectiveness in improving preventive care uptake rates from a population perspective.

With the macro perspective at the county level, this study delved deeper into racial and ethnic disparities in impact. Even though hospital–community partnerships are beneficial in general, the findings indicated that hospital–community partnerships disproportionately affect racial and ethnic minorities. In the White population, hospital–community partnerships were positively associated with mammography screening and flu vaccination rates. Notably, these positive effects were not observed in racial and ethnic minorities. Our finding provides counterpart evidence to the narratives that hospital–community partnerships can be beneficial to decrease health inequities. It highlights that while hospital–community partnerships can be effective, their benefits may be unevenly distributed among racial and ethnic minorities. The findings also align with previous studies indicating that, despite interventions such as free mobile mammography initiatives, racial and ethnic minorities still face care delays due to systematic access and cost barriers, unlike their White counterparts.^41^ These findings reflect the motivations behind hospitals’ partnership efforts with community organizations. While it is often assumed that hospital–community partnerships are determined by prevailing community needs (e.g., through CHNA),^13^ in fact, an existing study found that they are more likely driven by the social capital within the community.^37^ As hospitals are expected to expand their role beyond traditional patient care through these partnerships, resources and capacities associated with advantaged populations may further leverage the impact of partnerships on preventive care. Other studies have also found that safety-net hospitals are more likely to engage in hospital–community partnerships to leverage resources and expertise within their communities, but merely partnering with community organizations did not show any significant improvement in their vulnerable patients’ outcomes.^4^ Hospitals serving racial and ethnic minorities need more financial and managerial capabilities to address complex health needs. These hospitals also need stronger partnerships with grassroots community organizations to effectively tackle the root causes of health care inequality. Our findings underscore these additional needs to develop tailored care navigation, education, and coordination for minorities, aiming to reduce disparities in the impact of hospital–community partnerships. In addition, designing culturally competent interventions targeting minority populations through partnerships is also necessary to ensure their effectiveness.^42,43^

While this study contributes to the literature regarding the effectiveness of hospital–community partnerships on preventive care, there are several limitations that should be addressed in future studies. First, this study focused on the outsized effect of hospital–community partnerships at the county level, so the average of the common components among the nine different hospital–community partnerships within a county may be simplified. In addition, this study aimed to generalize the overall hospital–community partnerships with a macro perspective, which may compromise the extraction of meaningful information on hospital–community partnerships, such as breadth and depth of such partnerships.^37^ Future studies need to explore which community partners are most effective in improving health equity and what types of partnerships can create meaningful change for racial and ethnic minorities. Second, the measures for preventive health care interventions were available only at the county, not at the hospital service areas or hospital level, which restricted the analysis to the county level. Third, the preventive intervention measures in our analysis were based solely on Medicare enrollees, which limits our understanding of the impact of partnerships on preventive interventions to older adult population with Medicare health care coverage, excluding younger populations or those with other types of insurance. Lastly, the measures for preventive health interventions in the MMD Tool were not available in all counties across race and ethnic groups, limiting the study sample coverage. We also had to exclude AIAN and Asian groups due to the high number of missing observations for these groups. Despite these data limitations, the study sample covered about half of the counties in the United States, including all 50 states, and encompassed more than half of U.S. hospitals enrolled in the AHA. This provided a substantial dataset for exploring the effectiveness of hospital–community partnerships on preventive care from the macro perspective. Lastly, this study focused on pooled cross-sectional datasets in the pre-COVID period to explore the effectiveness of partnerships on preventive health care interventions, recognizing that preventive care measures, such as mammography screening and flu vaccination, can be significantly influenced by the COVID-19 pandemic. Future studies should broaden the scope of research to explore the outsized impact of COVID-19 in the linkage between hospital–community partnerships and preventive interventions. This expansion would enable an examination of how hospitals collaborate with community organizations to promote preventive care and reduce health disparities in the evolving health care landscape.

## Health Equity Implications

The findings provide important health equity policy and implications. In line with previous studies,^44,45^ the findings emphasize the necessity of designing hospital–community partnerships that specifically target racial and ethnic minorities to address health disparities. This is particularly crucial for the uptake of preventive interventions such as mammography screening and flu vaccinations, as racial and ethnic minorities have unique cultural norms, beliefs, systematic access challenges, and cost barriers that can contribute to medical mistrust.^32^ Our findings reveal that overall hospital–community partnerships, when not carefully tailored to address these differences across racial and ethnic groups, may disproportionately benefit only non-Hispanic White groups. Therefore, it is important to design multifaceted hospital–community partnerships that build community capacity to understand the needs of minorities^46^ and incorporate culturally competent interventions specifically talied to minority populations.^44,47^

## Abbreviations Used

ACA: Affordable Care Act
ACS: American Community Survey
AHA: American Hospital Association
AIAN: American Indian/Alaska Native
CHNA: Community Health Needs Assessment
CHRN: County Health Ranking National
DVs: Dependent Variables
FY: Fiscal Years
GLS: Generalized Least Squares
MMD: Mapping Medicare Disparities
OLS: Ordinary Least Squares
PCP: Primary Care Provider
